# #Fitmom: an experimental investigation of the effect of social media on body dissatisfaction and eating and physical activity intentions, attitudes, and behaviours among postpartum mothers

**DOI:** 10.1186/s12884-022-05089-w

**Published:** 2022-10-12

**Authors:** Lisa Tang, Marika Tiggemann, Jess Haines

**Affiliations:** 1 Department of Family Relations and Applied Nutrition, University of Guelph, 50 Stone Road East Guelph, N1G 2W1 ON Guelph, Canada; 2 School of Psychology, Flinders University, Adelaide, SA Australia

**Keywords:** Social media, Body dissatisfaction, Eating behaviour, Physical activity, Postpartum

## Abstract

**Background:**

Research has shown that body dissatisfaction is higher during the postpartum period compared to other periods of life, and strongly associated with disordered eating behaviours, which can lead to adverse health outcomes. While results from cross-sectional studies suggest that social media may play an important role in body dissatisfaction among postpartum mothers, causal inference is limited due to the observational nature of the existing research. The objective of this study is to experimentally test the effect of body-focused social media on the body dissatisfaction and eating and physical activity intentions, attitudes, and behaviours of postpartum mothers.

**Methods:**

Postpartum mothers of infants 0–6 months (n = 132) were randomly assigned to view either body-focused social media posts (n = 65), or a control set of infant feeding tips (n = 67). ANCOVA was used to examine differences between the intervention and control group on levels of body dissatisfaction, eating and physical activity intentions, attitudes, and behaviours. There were two follow-up time points, immediately post intervention and 1-month post intervention to measure potential sustained effects of intervention.

**Results:**

Exposure to body-focused social media posts resulted in higher levels of body dissatisfaction, mean difference 1.54 (p = 0.002); poorer body image, mean difference 0.41 (p = 0.007); eating attitudes, mean difference 2.26 (p = 0.025); and higher levels of restrained eating behaviours, mean difference 0.39 (p < 0.001) among the intervention group post intervention. Mothers in the intervention group also reported higher levels of inspiration to be active, mean difference 0.48 (p = 0.021) post intervention. A sustained effect was found for restrained eating, mean difference 2.03 (p < 0.001) and poorer eating attitude, mean difference 0.29 (p = 0.001) at 1-month follow-up. No sustained effects were found for any other outcomes at 1-month follow-up.

**Conclusion:**

Social media exposure to body-focused social media posts negatively affect postpartum mothers’ body dissatisfaction and health behaviours. Further experimental research that includes an interactive social media component is needed among this population.

**Trial Registration:**

NCT05181280, Study ID Number: 054798. Registered 06/01/2022. Retrospectively registered, https://clinicaltrials.gov/ct2/show/NCT05181280.

## Introduction

The World Health Organization describes the postpartum period as the most critical and yet most neglected phase in the lives of mothers [1]. The postpartum period is associated with significant changes to the weight, shape, and size of a woman’s body [2]. For many women, this is an adjustment that results in negative feelings toward their body [3, 4]. Research has shown that body dissatisfaction is higher during the postpartum period compared to other periods of life, and these feelings of body dissatisfaction are in a state of fluctuation during postpartum [4, 5]. Research by Coker and Abraham (2015) that followed body weight dissatisfaction levels of women through pregnancy up to 12 months postpartum found that body dissatisfaction steadily decreased as women progressed through their postpartum period with measures at 3, 6, and 12 months postpartum [5]. Specifically, body dissatisfaction returned to pre-pregnancy levels despite mothers not returning to their pre-pregnancy weight at 12 months postpartum [5], suggesting women progressively adapt and accept their changed postpartum body. However, a study by Clark et al. (2009) yielded more nuanced results. Specifically, women reported feeling the least strong and fit in early pregnancy, and although their feelings of fitness and strength returned to pre-pregnancy levels at 12 months postpartum, their self-perceptions of attractiveness remained unchanged through pregnancy and postpartum [6]. Similarly, a 2007 study among the perinatal population found that compared to pre-pregnancy and late-pregnancy, women reported higher levels of body dissatisfaction during the postpartum period, with 6 months postpartum being the time of highest reported body image concern [4].

Research has shown that body dissatisfaction is strongly associated with disordered eating behaviour [7]. Once disordered eating behaviours are established, longitudinal studies have shown them to continue for six to 10 years [8, 9]. This is a significant concern as disordered eating behaviours are associated with poor diet quality including an increase in the intake of calorically dense, nutrient poor foods [10], and adverse health outcomes including increased risk of depression, obesity, and developing a clinical eating disorder [8].

Appearance culture, social comparison, and the internalization of appearance ideals are strongly associated with body dissatisfaction among women [11–13]. Western body image ideals have been constructed through several societal influences, one of the most impactful being media. Substantial research has shown that media messages and images have a strong adverse influence on women’s body dissatisfaction and disordered eating behaviours [14, 15]. However, existing research has focused mainly on the influence of traditional forms of media, such as television and magazines, on women’s body dissatisfaction [16], and disordered eating behaviours [14]. Over the last decade, social media has replaced many traditional modes of media to become the predominant mode of communication. Indeed, the ubiquitous and mainstream nature of social media has changed the communication landscape, resulting in a gap in our understanding of current influences on body dissatisfaction and eating behaviour.

Previous research examining the effect of social media on body dissatisfaction found that body-focused messaging meant to inspire women to be fit and active referred to as “fitspiration” (a combination of the words fitness and inspiration) resulted in higher levels of inspiration to be active and eat healthy [17], but also greater body dissatisfaction among young women [17, 18]. Similarly, many social media posts targeted toward mothers such as “#fitmom” and others that show before and after pictures of postpartum weight loss are meant to inspire mothers to be fit and active. However, limited research has explored how these messages and images on social media networking platforms, such as Facebook, Twitter, and Instagram influence body dissatisfaction and disordered eating and physical activity attitudes and behaviours among women during the postpartum period. This is a critical gap because, although the number of women aged 18 and older using social media is high at 78% [19], it is even higher for mothers with nearly 90% using social media [20]. The few studies that have explored the impact of social media on body dissatisfaction during the postpartum period have used cross-sectional designs [21, 22]. Cross-sectional findings are limited in their ability to infer causality by possible differential recall of social media exposure, and the inability to determine the temporal order of the exposure and outcome This requires an experimental design. However, to date, no experimental studies investigating the impact of social media messaging on body dissatisfaction among postpartum mothers has been conducted.

To address this gap, we conducted an experimental study examining the impact of body-focused social media on body dissatisfaction among postpartum mothers. The primary objective of this study was to determine the extent to which body-focused social media, compared with the control condition, resulted in feelings of body dissatisfaction immediately following exposure and again at 1-month follow-up among postpartum mothers with children aged 0–6 months. Our secondary objectives were to determine the impact of body image messaging on postpartum mothers’ eating and physical activity intentions, attitudes, and behaviours.

## Methods

### Design

The Moms on Media Study (MOMS) is a randomized experiment designed to test the impact of social media messaging on body dissatisfaction, eating and physical activity attitudes and behaviours among postpartum mothers.

**Fig. 1:**
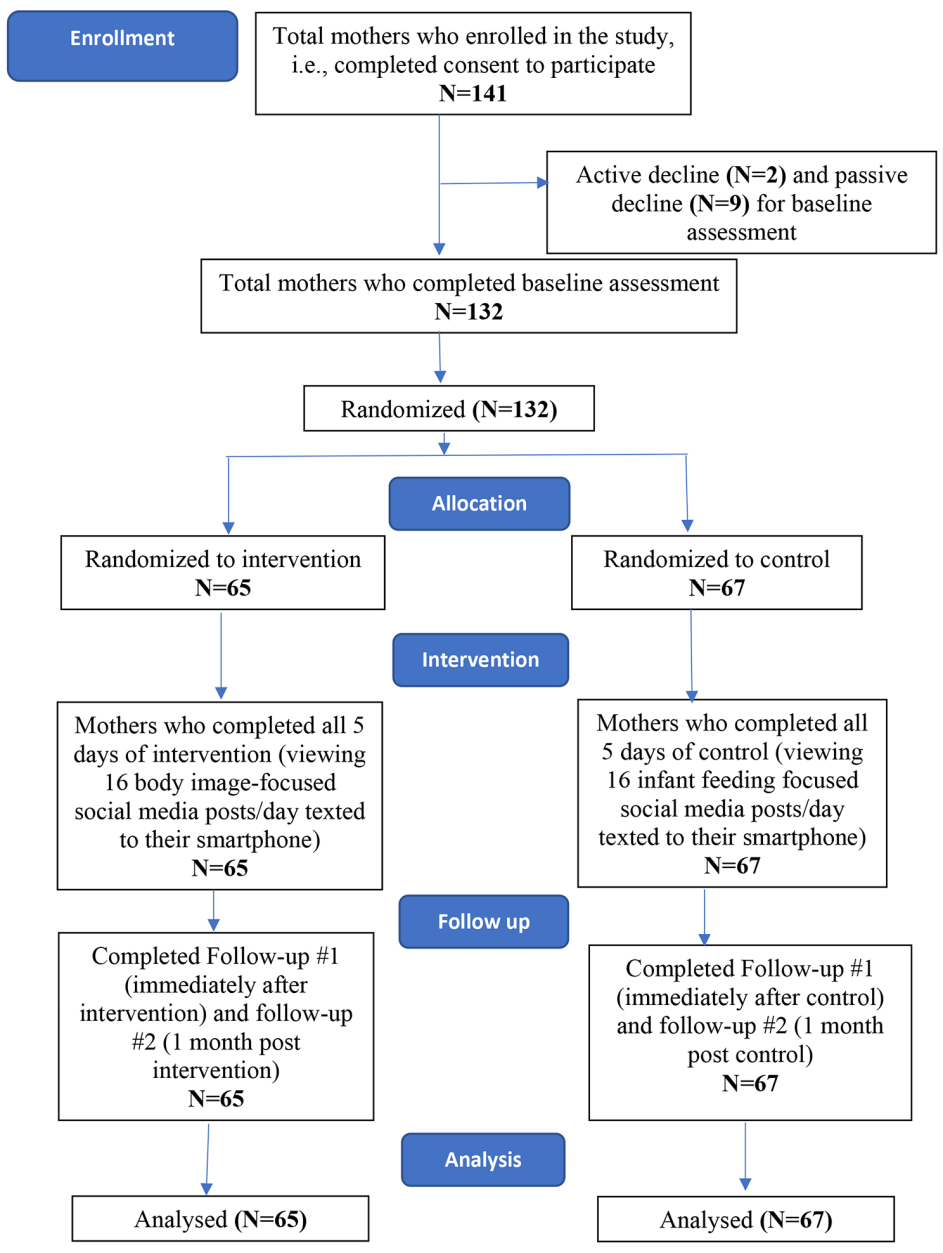
Participant involvement beginning from enrollment to final analytic sample for the Moms on Media Study

Between November 15 and November 24, 2020, the MOMS study enrolled 141 mothers of infants 0–6 months of age. Of those enrolled, 132 mothers completed their baseline assessment and were randomized using a random digit calculator into two groups: intervention (n = 65) or control (n = 67). All mothers received 15 social media posts each day over a 5-day period. These posts consisted of both the posted image and accompanying comment by the poster, as well as comments by those who viewed and responded to the post. Mothers randomized to the intervention received body image focused posts targeting mothers, and mothers randomized to the control received posts specific to infant feeding tips. All mothers completed a baseline survey, a follow-up survey immediately after the 5-day exposure period, and a final survey at 1-month follow-up (Fig. 1).

## Materials

### Stimuli

Two sets of 75 social media posts (15 posts/day over 5 days) were created for the study, one set for the intervention and one set for the control. In both sets, social media posts consisted of trending posts that were targeted toward mothers and were gathered from public profiles on Instagram, Facebook, TikTok, Twitter, and Snapchat. Social Media posts from TikTok included the original video that was posted on the TikTok platform. For the intervention group, social media posts included full body images/video of mothers with their infant child visible in most posts. Trending images were gathered by searching for hashtags related to mothering, body, and fitness (e.g., #fitmom, #3monthspostpartum, #momlife) on the various social media platforms.

The intervention images were selected from an initial pool of 120 images based upon ratings from five independent mothers of children under 2 years of age. Based on previous research by Tiggemann, Hayden, Brown, and Veldhuis (2018), each mother rated each image on the mother’s body size (1- extremely thin to 5- extremely overweight) and level of attractiveness (1- extremely attractive to 5- extremely unattractive). This scale was adapted to also include a rating on whether the social media post depicts the ideal image of motherhood (1- strongly agree to 5- strongly disagree). Based on previous research, images with a rating of ‘1’ or ‘2’ on thinness were considered as representative of the thin ideal, and images with rating of ‘3’ or ‘4’ were considered average [23]. The average rating for size, attractiveness, and ideal representation of motherhood was calculated for each image, and then summed to calculate a final numerical score for each image, with lower scores indicating closer to the ideal body and image of motherhood. The 75 images with the lowest total score were used for the intervention.

For the control arm of the study, social media posts included feeding tips for infants 0–6 months, including transitioning to solid foods. There were no images of mothers in any of the social media posts used for the control group. All images were reviewed by a Registered Dietitian to ensure the feeding tips were age-appropriate and in line with current infant feeding guidelines. Trending images were gathered by searching for social media posts created by public health and hashtags related to infant feeding (e.g., #babyfood, #infantfeeding, #firstfoods) on the various social media platforms.

## Measures

### Body dissatisfaction

Body dissatisfaction was measured using the body satisfaction subscale from the Multidimensional Eating Disorder Inventory [24], a 9-item subscale that measures satisfaction with physical appearance. This subscale includes questions such as “I think my stomach is too big”. This subscale has been validated among a diverse population of women [24–26], with good internal consistency [24]. Response options are “never”, “rarely”, “sometimes”, “often”, “usually”, and “always”, and coded as 1–6 respectively [24]. Higher scores indicate higher levels of body dissatisfaction [24].

### Body image

Body image was measured using the Body Image States Scale (BISS) [27], a 6-item scale that measures an individuals’ momentary evaluation of their body image. This questionnaire was found to have good internal consistency and has been evaluated among women (Cronbach’s Alpha 0.77) [27]. Each BISS question begins with “Right now I feel…” and has nine response options ranging from “E*xtremely satisfied”* to “*Extremely dissatisfied”* about how they feel toward their physical appearance, body shape, body weight, and physical attractiveness. Response options were scored from 1 to 9, with higher scores indicating lower body image.

### Healthy eating and physical activity inspiration

Following previous research from Tiggemann & Zaccardo (2015), two items were used to assess participants’ level of inspiration to be physically active and eat healthy on a 7-point Likert scale (1 = not at all inspired, 7 = very inspired) [17].

### Physical activity behaviour

Physical activity was measured using the International Physical Activity Questionnaire – Short Form (IPAQ-SF). The IPAQ-SF is a globally used physical activity questionnaire that prompts participants to report their vigorous (e.g., heavy lifting, running), moderate (e.g., bicycling at regular pace), and walking activity, as well as their time spent sitting over the last 7-day period [28, 29]. Following the IPAQ-SF protocol [28], responses were then calculated to obtain the Metabolic Equivalent of Task (MET) score to determine their level of activity.

### Eating attitudes

Eating attitudes were measured using Eating Attitudes Test (EAT-26), a 26-item scale that measures likelihood of having disordered attitudes toward eating [24]. This well-established scale has been validated among postpartum women with good internal consistency (Cronbach’s alpha 0.86) [30] EAT-26 includes questions such as “I am terrified about being overweight”, “I feel extremely guilty after I eat”, and “I engage in dieting behavior”. Response options include “never”, “rarely”, “sometimes”, “often”, “usually”, and “always”. Responses are scored as 0, 0, 0, 1, 2, 3, respectively. Scores are summed (minimum score 0, maximum score 78) with higher scores indicating greater disordered eating attitudes [31].

### Eating behaviour

Eating behaviour was measured using the Dutch Eating Behaviour Questionnaire (DEBQ), a 33-item questionnaire with 3 subscales that measures restrained eating, emotional eating, and external eating [32]. This well-established questionnaire was found to have good internal consistency among each of the 3 subscales (Cronbach’s Alpha 0.80–0.95), has been used to examine the eating behaviours of mothers [33], and postpartum mothers [4], and has been validated among diverse populations [34, 35]. The DEBQ includes questions such as “how often do you refuse food and drink offered because you are concerned about your weight?”. Response options range from never to very often and are assigned a score between 1 and 5. Scores were summed for each of the subscales, with higher scores indicating more restrained eating, more emotional eating, and more external eating [32].

### Demographic information and social media use

Participant date of birth, income, education level, height, and weight, were self reported. Mother BMI was calculated using their self-reported height and weight. Mother age was collected by calculating the difference between their reported date of birth and date the baseline survey was completed. Child age in months and weeks was reported by the mother. Social media use was measured two ways: the *volume* of social media use and the *frequency* of social media use. To measure volume, participants were asked to report the amount of time per day spent on each social media platform, specifically, Instagram, Snapchat, Twitter, Facebook, TikTok, Pinterest, Tumblr, Reddit, Linkedin and other platform not listed. Responses were summed to determine how much time per day each participant spent on social media. To measure frequency, participants were asked how often they checked each of the social media platforms listed above. Options were “I do not use this platform”, “less than once a week”, “1–2 days a week”, “3–6 days a week”, “once a day”, “2–4 times a day”, “5 or more times a day”. Responses were coded as 0, 0.5, 1.5, 4.5, 7, 21, 35 and summed to get total number of social media checks per week.

### Procedure

Following approval from the University of Guelph Research Ethics Board, participants were recruited for the Moms on Media Study (MOMS) through social media platforms including Twitter and Facebook. Advertisements on social media included a link to the MOMS recruitment website that provided the study details. Interested participants completed an online eligibility questionnaire and provided informed consent. Eligible participants were those who could respond to surveys in English, have a child aged 0–6 months, have not been diagnosed with anxiety or depression by a physician, and own a smartphone. Participants completed a baseline questionnaire and were then randomized into one of two groups: intervention (N = 65) or control (N = 67). This study was approved by the University of Guelph Research Ethics Board and followed the Consolidated Standards of Reporting Trials (CONSORT) reporting guidelines for parallel group randomized controlled trials.

Baseline and follow-up surveys were emailed to participants and completed online, and delivery of the intervention and control exposure was sent via text to participants. Based on previous social media and body image research [23, 36–38], participants in both the intervention and control condition had 1 exposure session consisting of 15 social media posts per day over 5 days. Participants received a text to their smartphone with a link to the online program Qualtrics© where they scrolled through social media posts in a way that mimicked a real experience on a social media platform. To ensure participants attended to the images, a timer was set in Qualtrics© that required images to be viewed for a minimum of 2 min and 25 s before ending their exposure session. For the intervention group, each exposure session consisted of 15 ideal-image body-focused posts targeting mothers. For the control group, social media posts consisted of infant feeding tips. Participants then completed a follow-up survey immediately after the 5-day exposure period (post-intervention) and at 1-month follow-up. Participants received a grocery gift card for their participation.

### Statistical analysis

The sample size calculation completed for this study was based on the expected detectable difference of 4.0 (SD = 1.5) in body dissatisfaction between groups. Results from previous experimental research with young women found a similar difference in body dissatisfaction [17] demonstrating that such an effect is plausible for our study. Based on a hypothesized detectable difference of 4.0, with an alpha of 0.05 (two-sided), 80% power, it was estimated that a sample size of 115 participants was needed. This calculation was based on two-sample t tests, which would provide a conservative estimate as an ANCOVA is more efficient than t tests. We aimed to recruit approximately 130 to allow for possible attrition over the study period.

RStudio, version 2021.09.0, was used to perform all analyses and run descriptive statistics to describe population age, socioeconomic status, and BMI. ANCOVA was used to determine whether a significant different exists between the intervention and control groups on measures of healthy eating and physical activity inspiration, physical activity behaviour, eating attitudes and behaviour, body dissatisfaction, and body image, while adjusting for baseline scores. Statistical significance was defined as p < 0.05.

## Results

### Participant characteristics

The mothers in the sample were between 23 and 40 years of age with a mean age of 32.5 (SD = 3.6). Their mean Body Mass Index (BMI) was 26.7 (7.6), mean number of children was 1.7 (0.9), and mean baby age was 3.4 (1.7) months. Participant demographics can be found in Table 1. Mothers spent an average of 4.8 (3.2) hours on social media each day and checked social media an average of 10 (3.9) times per day.

**Table 1:** Characteristics of mothers who participated in the Moms on Media Study, N = 132

Mother Characteristic	N = 132
Age, mean (SD)	32.5 (3.6)
Weight status, BMI, mean (SD)	28.7 (7.6)
Number of children, mean (SD)	1.7 (0.9)
Baby age in months, mean (SD)	3.4 (1.7)
Income, n (%)	
<$40,000	17 (12.9)
$40,000-$69,999	32 (24.3)
$70,000-$99,999	48 (36.3)
$100,000-$149,999	16 (12.1)
>$150,000	7 (5.3)
Did not answer	12 (9.1)
Maternal Education, n(%)	
Low ( < = secondary school)	3 (2.3)
Medium (college, some university, or technical school)	37 (28)
High (university degree +)	91 (68.9)
Prefer not to answer	1 (0.8)
Ethnicity, n(%)	
White	117 (88.6)
Other	15 (11.4)
Time on Social Media, hrs/day, mean (SD)	4.8 (3.2)
Social Media checks/day, mean (SD)	9.9 (3.9)

*Age had an N = 130, weight status had an N = 129, and number of children had an N = 131

One-way ANOVAs showed that the intervention and control groups did not differ in mean age, income, BMI, infant age, number of children, or social media use. There were also no significant differences between the two groups at baseline on measures of emotional eating, external eating, body dissatisfaction, eating attitudes, and body image. However, differences at baseline between the two groups were found for inspired to be physically active, mean difference 0.52 (p = 0.04); inspired to eat healthy, mean difference 0.55 (p = 0.02); and restrained eating, mean difference 0.33 (p = 0.01). Analysis of covariance was used to control for differences at baseline by adjusting the participants follow up score for their baseline score to ensure results are not impacted by the baseline imbalance.[39] Baseline means and adjusted means (controlling for baseline) for post-intervention and 1-month follow-up can be found in Table 2.

**Table 2:** Baseline mean scores (SD), and adjusted mean score (SD) for post-intervention and 1-month follow-up for the Moms on Media Study

	Baseline		Post-Intervention		1-month Follow-up	
	**Control**	**Intervention**	**Control**	**Intervention**	**Control**	**Intervention**
	M(SD)	M(SD)	M(SD)	M(SD)	M(SD)	M(SD)
**Body dissatisfaction**	10.36 (6.12)	11.52 (7.07)	**9.64 (5.93)**	11.18 (6.91)*	8.48 (5.72)	9.63 (7.05)
**Body Image**	5.70 (1.49)	5.89 (1.5)	**5.52 (1.66)**	5.93 (1.63)*	5.65 (1.6)	5.75 (1.72)
**Inspired to be active**	**4.96 (1.51)**	**5.48 (1.3)**	**4.87 (1.34)**	5.35 (1.36)*	5.19 (1.14)	5.51 (1.23)
**Inspired to eat healthy**	**4.87 (1.4)**	**5.42 (1.26)**	5.03 (1.11)	5.37 (1.44)	5.37 (0.90)	5.55 (1.15)
**Physical activity**	1737 (1814)	1886 (1645)	1675 (1818)	1836 (1835)	1632 (2637)	2244 (2371)
**Eating Attitude Test**	6.55 (12.67)	6.26 (6.13)	**4.02 (3.50)**	6.28 (8.13)*	**3.97 (3.98)**	6.00 (7.39)**
**Restrained eating**	**2.23 (0.71)**	**2.56 (0.73)**	**2.21 (0.72)**	2.6 (0.76)**	**2.24 (0.68)**	2.53 (0.76)**
**Emotional eating**	2.74 (0.90)	2.76 (0.94)	2.65 (0.92)	2.66 (0.91)	2.65 (0.89)	2.69 (0.95)
**External eating**	3.27 (0.41)	3.24 (0.55)	3.2 (0.43)	3.17 (0.46)	3.22 (0.43)	3.16 (0.54)

^a^ Mean score at post-intervention and 1-month follow-up adjusted for baseline score

*p < 0.05

**p < 0.001

### Effect of body-focused social media on body dissatisfaction and body image

ANCOVA results show that body dissatisfaction was significantly higher for the intervention group compared to the control group post-intervention, mean difference 1.54 (p = 0.002). Similarly, body image was significantly higher for the intervention group compared to control at post-intervention with a mean difference of 0.41 (p = 0.007). These differences in body dissatisfaction and body image between the two groups were not sustained at 1-month follow-up. (Table 2).

### Effect of body-focused social media on physical activity and healthy eating inspiration

ANCOVA results show that at post-intervention mothers who viewed the body-focused posts scored significantly higher for inspired to be physically active compared to the control group, mean difference 0.48 (p = 0.021). Differences between these measures of inspiration to be active were not sustained at 1-month follow-up. No significant differences were found for inspired to eat healthy between the two groups post-intervention or at 1-month follow-up. (Table 2)

### Effect of body-focused social media on activity levels

No significant differences were found for physical activity between the intervention and control group at either post-intervention or 1-month follow-up. (Table 2)

### Effect of body-focused social media on eating attitude

ANCOVA results show that poor eating attitudes were significantly higher for the intervention group compared to the control group post-intervention with a mean difference of 2.26 (p = 0.025). This significant difference between the two groups was sustained at 1-month follow-up with a mean difference of 2.03 (p = 0.001). (Table 2).

### Effect of body-focused social media on restrained, emotional, and external eating

ANCOVA results show that restrained eating was significantly higher for the intervention group compared to the control group post-intervention, mean difference = 0.39 (p  < 0.001). This difference between the two groups was sustained at 1-month follow-up with a mean difference of 0.29 (p  < 0.001). No significant differences were found between the intervention and control group for emotional or external eating at post-intervention or 1-month follow up. (Table 2)

## Discussion

The primary objective of this study was to experimentally test the effect of body image messaging targeting mothers on feelings of body dissatisfaction immediately following exposure and again at 1-month follow up. Overall, this study has extended the experimental investigation of the impact of social media on body dissatisfaction by investigating the effect of social media on an important segment of the population, specifically, postpartum mothers.

Our findings revealed that while scores for body dissatisfaction and body image improved over the study period for mothers in the control group, scores for body dissatisfaction and body image remained relatively stable among mothers in the intervention group. This resulted in a significant difference in these two outcomes at post-intervention among the intervention and control group. Previous observational research by Coker and Abraham (2015) found that new mothers’ body dissatisfaction slowly improved across the postpartum period until it reached pre-pregnancy levels at approximately 12 months postpartum [5]. Our finding that this improvement in body dissatisfaction appeared to happen among mothers in our control group, but not for mothers in our intervention group, suggests that exposure to body-focused social media prevented intervention mothers from the progression of feeling better about their bodies as they move further from birth toward 12 months postpartum. At 1-month follow-up, differences between the control and intervention group were not sustained and mean scores for body dissatisfaction decreased in both groups, which suggests that when postpartum mothers no longer view ideal body-focused posts on social media, their levels of body dissatisfaction may return to the natural progression toward improvement over time.

Although no significant differences were found between the intervention and control group for emotional or external eating at either time point, restrained eating was higher among the intervention group post-intervention. Similarly, the intervention group had significantly poorer eating attitudes compared to the control group post-intervention. Our findings are consistent with and build upon previous research identifying that viewing ideal social media images is linked to restrained eating [40], and disordered eating attitudes among young women [41]. Indeed, this study focused on the effect of ideal body image posts on social media on body image and health behaviour, but positive social media messaging may yield similar but opposite results. For example, recent research has suggested that reliable health information posted to social media may positively predict desired health behaviours among a population [42]. Future research should focus on examining whether positive messaging and health information may interrupt the potentially harmful content typically posted to social media.

The significant differences found in the present study between the control and intervention group for restrained eating and eating attitude were sustained at 1-month follow-up. This points toward the possibility that viewing body-image content on social media may have a more robust impact on eating behaviours as compared to feelings of inspiration and body image. One possible reason for this is that research has shown poor dietary behaviours are difficult to change [43]. Thus, dietary behaviour may be more entrenched than feelings and could potentially explain the difference found in the 1-month follow up among restrained eating versus feelings of inspiration. Further research is needed to confirm these findings and identify the mechanisms behind these differences.

Compared to the control group, feeling inspired to be physically active was higher among those in the intervention group at post-intervention. This is consistent with previous research that found viewing fitspiration images on social media led to higher levels of inspiration to improve fitness [17, 18]. Our results show that body-focused messaging such as “#fitmom” and before and after images of postpartum weight loss appear to inspire postpartum mothers to be physically active. However, where our findings differ from previous research [17] is that body-focused social media posts did not have a significant effect on postpartum mothers’ inspiration to eat healthy. Interestingly, these body-focused social media posts also did not result in any change in physical activity behaviour, which has been shown in other social media research [18, 44]. Taken together, although body-focused social media images may increase inspiration toward being physically active, they may not increase actual physical activity but instead increase body dissatisfaction and disordered eating behaviour, resulting in an overall negative effect on women.

Strengths of our study include the experimental design that provided participants with social media posts directly to their smartphone in a way that mimicked the common experience of scrolling through social media posts. This may have provided a more realistic social media experience when compared to social media experiments done in a lab setting as they were able to view study images in the same environment where they would normally view their own social media. Second, this study provided posts from a variety of social media platforms, including embedded video and images as well as comments, to closely align with the multi-media experience of the current social media environment. Finally, the focus on postpartum mothers helps to fill a key gap in our understanding of how social media impacts mothers’ body dissatisfaction and associated eating and physical activity attitude and behaviours.

This study had some limitations that should be considered when interpreting the results. First, participants were predominantly white, middle-class income earners, and all participants were Canadian, and therefore may not be generalizable to the broader population or outside of Canada. Future research could focus on recruiting a more ethnically, racially, and socioeconomically diverse population to increase generalizability. Second, there was no ability to interact (e.g., like or comment) with the social media posts, which is a key aspect in distinguishing social media from traditional forms of media [45]. Future social media experimental research in this area should include an interaction component in their intervention condition. Third, this study took place during the COVID-19 pandemic and therefore participants may have social media habits that are specific to being isolated at home during a pandemic. Finally, there may be differences in the way mothers internalize ideal media images. For example, previous research has shown that internalization of the ideal body acts as a mediator between appearance-focused social comparison and body dissatisfaction [46] and a moderator for the effect of weight stigma on exercise avoidance [47]. Future research is needed to further examine these relationships among postpartum mothers.

The results of this study provide a more in-depth understanding of the effect of body-focused social media posts on the body dissatisfaction and eating and physical activity attitudes and behaviours of postpartum mothers. It is important that we continue to research social media and body image among this population as body dissatisfaction among mothers not only affects the health of mothers, but can also negatively influence the eating attitudes [48] and behaviours [49] of their children [50]. Findings from this study help equip healthcare providers with research-based evidence to support the development of social media literacy interventions, including how to engage positively with social media to reduce ideal body-focused images, within prenatal and postpartum care. Specifically, including social media literacy within the healthcare environment at prenatal classes and postpartum care appointments with the family doctor and other healthcare providers may help to improve body dissatisfaction and associated health behaviours among postpartum mothers.

## Conclusion

Overall, body-focused social media posts had an effect on postpartum mothers’ levels of body dissatisfaction, physical activity inspiration, eating attitudes and behaviours. Given that mothers’ use of social media is high, with nearly 90% of mothers regularly using social media [20], fully understanding the influence of social media engagement on postpartum mothers’ health behaviours is critical. This knowledge will help guide healthcare interventions, equipping healthcare professionals with research-based evidence to support postpartum mothers in maintaining positive body image and healthy diet and exercise behaviours during the postpartum period. Further study is needed that includes an interactive component to the social media experimental intervention and a more ethnically and socioeconomically diverse population.

## Data Availability

The datasets used and/or analysed during the current study are available from the corresponding author on reasonable request.

## Declarations

All manuscripts must contain the following sections under the heading ‘Declarations’:

## List of Abbreviations

ANOVA: Analysis of variance
ANCOVA: Analysis of covariance.
BISS: Body Image States Scale.
BMI: Body Mass Index.
CONSORT: Consolidated Standards of Reporting Trials.
COVID-19: Coronavirus disease of 2019.
DEBQ: Dutch Eating Behaviour Questionnaire.
EAT-26: Eating Attitude Test (26 item scale).
IPAQ-SF: Physical Activity Questionnaire – Short Form.
MET: Metabolic Equivalent of Task.
MOMS: Moms on Media Study.
